# MHD convective heat transfer in a discretely heated square cavity with conductive inner block using two-phase nanofluid model

**DOI:** 10.1038/s41598-018-25749-2

**Published:** 2018-05-09

**Authors:** A. I. Alsabery, M. A. Sheremet, A. J. Chamkha, I. Hashim

**Affiliations:** 1 Refrigeration & Air-conditioning Technical Engineering Department, The Islamic University, Najaf, Iraq; 2 School of Mathematical Sciences, Faculty of Science & Technology, Universiti Kebangsaan Malaysia, 43600 UKM Bangi, Selangor Malaysia; 3 Department of Theoretical Mechanics, Tomsk State University, 634050 Tomsk, Russia; 4 Institute of Power Engineering, Tomsk Polytechnic University, 634050 Tomsk, Russia; 5 Department of Mechanical Engineering, Prince Sultan Endowment for Energy and Environment, Prince Mohammad Bin Fahd University, Al Khobar, 31952 Saudi Arabia; 6 RAK Research and Innovation Center, American University of Ras Al Khaimah, P.O. Box, 10021 Ras Al Khaimah, United Arab Emirates

## Abstract

The problem of steady, laminar natural convection in a discretely heated and cooled square cavity filled by an alumina/water nanofluid with a centered heat-conducting solid block under the effects of inclined uniform magnetic field, Brownian diffusion and thermophoresis is studied numerically by using the finite difference method. Isothermal heaters and coolers are placed along the vertical walls and the bottom horizontal wall, while the upper horizontal wall is kept adiabatic. Water-based nanofluids with alumina nanoparticles are chosen for investigation. The governing parameters of this study are the Rayleigh number (10^3^ ≤ *Ra* ≤ 10^6^), the Hartmann number (0 ≤ *Ha* ≤ 50), thermal conductivity ratio (0.28 ≤ *k*_*w*_ ≤ 16), centered solid block size (0.1 ≤ *D* ≤ 0.7) and the nanoparticles volume fraction (0 ≤ *ϕ* ≤ 0.04). The developed computational code is validated comprehensively using the grid independency test and numerical and experimental data of other authors. The obtained results reveal that the effects of the thermal conductivity ratio, centered solid block size and the nanoparticles volume fraction are non-linear for the heat transfer rate. Therefore, it is possible to find optimal parameters for the heat transfer enhancement in dependence on the considered system. Moreover, high values of the Rayleigh number and nanoparticles volume fraction characterize homogeneous distributions of nanoparticles inside the cavity. High concentration of nanoparticles can be found near the centered solid block where thermal plumes from the local heaters interact.

## Introduction

Natural convection heat transfer in cavities is a significant phenomenon in engineering systems and important applications in operations of solar collectors, cooling of containment buildings, room ventilation, heat exchangers, storage tanks, double pane windows, etc. A comprehensive review on natural convection in cavities was made by Ostrach^1^. Also, the problem of natural convection in cavities with discrete heat sources has important applications in electronic packaging, cooling of nuclear reactors, ignition of solid fuels^2,3^. Kaluri and Basak^4^ and Kaluri and Basak^3^ considered the problem of natural convection in a discretely heated square porous cavity filled with pure fluid. They used the finite element method for solving the governing equations together with the boundary conditions and the found that the methodology of the distributed heating with multiple heat sources can be considered as an effective strategy for the optimal thermal processing of materials. The thermal conductivity of nanoparticles is higher than that of traditional fluids. Thus, nanofluids can be used in a large industrial applications such as oil industry, nuclear reactor coolants, solar cells, construction, electronics, renewable energy and many others. Also, nanoparticles are used because they stay in suspension longer than larger particles. Thus, nanofluids can be used in a large industrial applications such as oil industry, nuclear reactor coolants, solar cells, construction, electronics, renewable energy and many others. A nanofluid as a working medium has been considered by many researchers for the simple reason that it has the presence of nanoparticles resulting in higher thermal conductivity of medium and the heat transfer becoming enhanced.

Khanafer *et al*.^5^ reported a problem of natural convective heat transfer in cavities partially occupied by nanofluids. Two approaches based on conservation equations have been adopted in the literature to investigate the numerical simulation heat transfer of nanofluids: single-phase model (homogenous) and two phase model^6^. The single-phase approach considers the fluid phase and the nanoparticles as being in thermal equilibrium where the slip velocity between the base fluid and the nanoparticles is negligible. On the other hand, the two-phase approach assumes that the relative velocity between the fluid phase and the nanoparticles may not be zero where the continuity, momentum and energy equations of the nanoparticles and the base fluid are handled using different methods. There are number of numerical studies used the single-phase model for simulation of the nanofluids. Hu *et al*.^7^ studied experimentally and numerically the natural convection heat transfer in a square cavity filled with TiO_2_–water nanofluids. They found that the average Nusselt number increased with the addition of nanoparticles. Sheikholeslami *et al*.^8^ conducted an experimental investigation on the enhancement of the heat transfer and pressure drop through a concentration of refrigerant-based nanofluid. Sheremet *et al*.^9^ and Alsabery *et al*.^10^ numerically investigated the natural convection heat transfer of nanofluid flow in different geometries. Recently, Alsabery *et al*.^11^ numerically considered the problem of natural convection heat transfer in an inclined square cavity using the nanofluid single phase model. They found that the heat transfer rate was enhanced with the increment of the nanoparticles volume fraction. Most of the above studies are used the Maxwell-Garnett and Brinkman models to estimate the effective thermal conductivity and viscosity of the nanofluid. Sheikholeslami and Seyednezhad^12^ studied the influence of electric field on nanofluid flow and natural convection in a porous media using CVFEM. However, the study of Corcione^13^ questions the validity of these models and tended to proposed a new models for estimating the effective thermal conductivity and viscosity of the nanofluid which appeared to be close to the experimental data. The results showed that the heat transfer rate enhanced with the relative concentration of nanofluid. The experimental study of Wen and Ding^14^ found that the slip velocity between the base fluid and particles may not be zero. Thus, the two-phase nanofluid model observed to be more accurate. Buongiorno^15^ proposed a non-homogeneous equilibrium model with the consideration of the effect of the Brownian diffusion and thermophoresis as two important primary slip mechanisms in nanofluid. Hamid *et al*.^16^ used the Buongiorno model to study Non-alignment stagnation-point flow of a nanofluid past a permeable stretching/shrinking sheet. Sheikholeslami *et al*.^17^ used the two-phase model of the nanofluid to investigate the thermal management for natural convection heat transfer in a 2D cavity. Garoosi *et al*.^18^ studied mixed convection heat transfer where the two-phase mixture model used to simulate the nanofluid in a two-sided lid-driven cavity with several pairs of heaters and coolers (HACs). Garoosi *et al*.^19^ used Corcione et al. model^13^ for the effective thermal conductivity and viscosity of the nanofluid to study numerically the problem of natural convection heat transfer in a heat exchanger filled with nanofluids. Very recently, Motlagh and Soltanipour^20^ investigated numerically the problem of natural convection of nanofluids in a square cavity using the two phase model. The results of these studies indicated that the heat transfer rate enhanced with the increasing of the concentration of the nanoparticles up to 0.04.

Recently the effect of the magnetic field on convective heat transfer in cavities were considered extensively due to its wide applications such as in the polymer industry, coolers of nuclear reactors, MEMs, purification of molten metals and many other important applications which can be used to control the convection inside cavities^21,22^ Pirmohammadi and Ghassemi^23^ investigated numerically the effect of the magnetic field on a steady laminar natural convection flow in an inclined square cavity. Mahmoudi *et al*.^24^ analysed numerically the magnetic field effect on natural convection in a two-dimensional cavity filled with nanofluid. They found that the presence of magnetic field tended to decrease the convection heat transfer. Ghasemi *et al*.^25^ considered the influence of the magnetic field on natural convection in a square cavity filled with Al_2_O_3_-water nanofluid. They concluded that the enhancement or deterioration of the convection heat transfer by the increasing of the solid volume fraction was clearly depending on the values of Hartmann and Rayleigh numbers. Using lattice Boltzmann method, Kefayati^26^ studied the effect of a magnetic field on natural convection in an open square cavity filled with water/alumina nanofluid where his results showed that the heat transfer decreased with the increment of Hartmann number and for various values of Rayleigh numbers and volume fractions. Sheikholeslami *et al*.^27^ used the KKL model of the nanofluid to investigate the MHD effects on natural convection heat transfer in a 2D cavity filled with Al_2_O_3_-water nanofluid using the lattice Boltzmann method. Sheikholeslami *et al*.^28^ used the same method to investigated the magnetic field effect on CuO-water nanofluid flow and heat transfer in a cavity. They considered in their study the effect of the Brownian motion on the effective thermal conductivity and they found that the enhancement in heat transfer increased as Hartmann number increase while it decreased with the increasing of Rayleigh number. Selimefendigil and Öztop^29^ used the finite element method to study the magnetic field and internal heat generation effects on natural convection in a square cavity filled with nanofluid and having different shaped obstacles. They found that the heat transfer rate was deteriorated with the presence of the solid obstacles. Recently, Sheikholeslami and Shehzad^30^ numerically reported the effect of external magnetic source on natural convection in a permeable media filled with Fe_3_O_4_-H_2_O nanofluid. Using the CVFEM simulation on the problem of nanofluid migration and convective heat transfer in a 2D porous cavity with an external magnetic filed was considered by Sheikholeslami and Shehzad^31^.

Sheikholeslami and Shehzad^31^ Sivaraj and Sheremet^32^ considered the influence of the applied magnetic field on natural convection in an inclined square porous cavity with a heat conducting solid block. Their results indicated that the inclusion of the magnetic field decreased the heat transfer rate within the square cavity. Sheikholeslami and Rokni^33^ and Sheikholeslami^34^ investigated the Brownian motion effects on the magnetic nanofluid flow and heat transfer in a 2D porous cavity using the CVFEM modeling. They concluded that the convective flow was a reducing function of the rising of Hartmann number.

Conjugate heat transfer (CHT) for a regular fluid has very important practical engineering applications in frosting practicalities and refrigeration of the hot obtrusion in a geological framing. For example, modernistic construction of thermal insulators which are formed of two diverse thermal conductivities (solid and fibrous) materials can be modeled by the partition length and conductivity model. Kim and Viskanta^35^ reported a conjugate convection in a differentially-heated vertical rectangular cavity filled with viscous (pure) fluids surrounded by four conducting walls. House *et al*.^36^ studied the natural convective heat transfer in a square cavity with a centred heat-conducting body and they found that the heat transfer reduced with an increasing of the length of the solid body. Ha *et al*.^37^ considered the effect of a centred heat-conducting body on unsteady natural convection heat transfer in a vertical cavities. Zhao *et al*.^38^ studied the effect of a centred heat-conducting body on the conjugate natural convection heat transfer in a square enclosure. The results show that the thermal conductivity ratio has strong influence on the flow within the square cavity. Mahmoodi and Sebdani^39^ used the finite volume method to investigate the conjugate natural convective heat transfer in a square cavity filled with nanofluid and containing a solid square block at the center. They concluded that the heat transfer rate decreased with an increasing of the size of the inner block for low Rayleigh numbers and increased at high Rayleigh numbers. Mahapatra *et al*.^40^ numerically used the finite volume method to investigate the CHT and entropy generation in a square cavity in the presence of adiabatic and isothermal blocks. They found that the heat transfer enhanced with the low Rayleigh numbers and for a critical block sizes. Alsabery *et al*.^41^ used the finite deference method to study the unsteady natural convective heat transfer in nanofluid-saturated porous square cavity with a concentric solid insert and sinusoidal boundary condition. Very recently, Garoosi and Rashidi^42^ used the finite volume method to investigate the two phase model of conjugate natural convection of the nanofluid in a partitioned heat exchanger containing several conducting obstacles. They found that the heat transfer rate was significantly influenced by changing the orientation of the conductive partition from vertical to horizontal mode.

The effect of the magnetic field on natural convection in a discretely heated square cavity with a conductive inner block has not been investigated yet. Therefore, the aim of this comprehensive numerical study is to investigate the MHD natural convection of Al_2_O_3_-water nanofluid in a discretely heated square cavity with conductive inner block using Buongiorno’s two-phase model. The authors of the present study believe that this work is a good contribution for improving the thermal performance and the heat transfer enhancement in some engineering instruments.

## Mathematical Formulation

**Figure 1:**
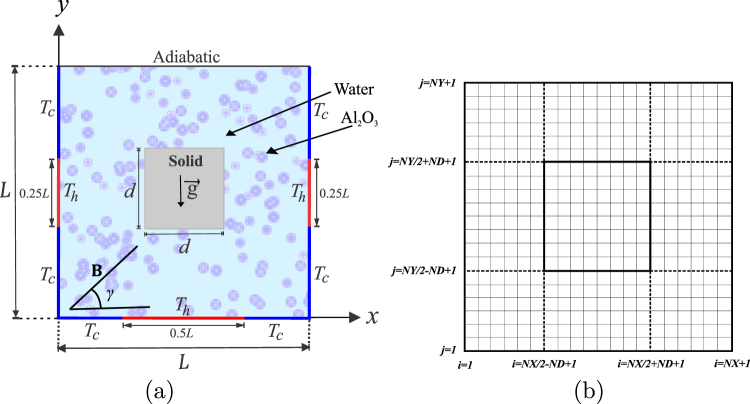
(**a**) Physical model of convection in a square cavity, and (**b**) grid-points distribution in the adiabatic inner block (*NY*/2 − *ND* + 1 ≤ *j* ≤ *NY*/2 + *ND* + 1, *NX*/2 − *ND* + 1 ≤ *i* ≤ *NX*/2 + *ND* + 1).

The steady two-dimensional natural convection problem in a square cavity with length *L* and with the cavity center inserted by a solid square with side *d*, as illustrated in Fig. 1. The Rayleigh number range chosen in the study keeps the nanofluid flow incompressible and laminar. Isothermal heat sources are shown by a thick red lines and the remaining parts are maintained at cold isothermal which represented by a thick blue lines. While the top horizontal wall is kept adiabatic. The boundaries of the annulus are assumed to be impermeable, the fluid within the cavity is a water-based nanofluid having Al_2_O_3_ nanoparticles. The Boussinesq approximation is applicable, the nanofluid physical properties are constant except for the density. By considering these assumptions, the continuity, momentum and energy equations for the laminar and steady state natural convection can be written as follows:

1 $$\nabla \cdot {\bf{v}}=0,$$

2 $${\rho }_{nf}{\bf{v}}\cdot \nabla {\bf{v}}=-\,\nabla p+\nabla \cdot {\mu }_{nf}\nabla {\bf{v}}+{(\rho \beta )}_{nf}(T-{T}_{c})\overrightarrow{g}+{\sigma }_{nf}{\bf{v}}\times \overrightarrow{{\bf{B}}},$$

3 $${(\rho {C}_{p})}_{nf}{\bf{v}}\cdot \nabla {T}_{nf}=-\,\nabla \cdot {k}_{nf}\nabla {T}_{nf}-{C}_{p,p}{J}_{p}\cdot \nabla {T}_{nf},$$

4 $${\bf{v}}\cdot \nabla \phi =-\,\frac{1}{{\rho }_{p}}\nabla \cdot {J}_{p},$$

The energy equation of the inner solid wall is

5 $${\nabla }^{2}{T}_{w}=\mathrm{0,}$$

where **v** is the velocity vector, $\overrightarrow{g}$ is the gravitational acceleration vector, $\overrightarrow{{\bf{B}}}$ represents the applied magnetic field, *φ* is the local volume fraction of nanoparticles and *J*_*p*_ is the nanoparticles mass flux. The subscripts *f*, *nf*, *p* and *w* represent the base fluid, nanofluid, solid nanoparticles and solid inner wall, respectively. Based on Buongiorno’s model nanoparticles mass flux can be written as:

6 $${J}_{p}={J}_{p,B}+{J}_{p,T},$$

7 $${J}_{p,B}=-\,{\rho }_{p}{D}_{B}\nabla \phi ,\,{D}_{B}=\frac{{k}_{b}T}{3\pi {\mu }_{f}{d}_{p}},$$

8 $${J}_{p,T}=-\,{\rho }_{p}{D}_{T}\nabla T,\,{D}_{T}=0.26\frac{{k}_{f}}{2{k}_{f}+{k}_{p}}\frac{{\mu }_{f}}{{\rho }_{f}T}\phi $$

where *D*_*B*_ and *D*_*T*_ are the brownian diffusion coefficient and the thermophoretic diffusivity coefficient. The thermo-physical properties of the nanofluid can be determined as follows:

The heat capacitance of the nanofluids (*ρC*_*p*_)_*nf*_ given is

9 $${(\rho {C}_{p})}_{nf}=(1-\phi ){(\rho {C}_{p})}_{f}+\phi {(\rho {C}_{p})}_{p}.$$

The effective thermal diffusivity of the nanofluids *α*_*nf*_ is given as

10 $${\alpha }_{nf}=\frac{{k}_{nf}}{{(\rho {C}_{p})}_{nf}}.$$

The effective density of the nanofluids *ρ*_*nf*_ is given as

11 $${\rho }_{nf}=(1-\phi ){\rho }_{f}+\phi {\rho }_{p}.$$

The thermal expansion coefficient of the nanofluids *β*_*nf*_ can be determined by:

12 $${(\rho \beta )}_{nf}=(1-\phi ){(\rho \beta )}_{f}+\phi {(\rho \beta )}_{p}.$$

The dynamic viscosity ratio of water-Al_2_O_3_ nanofluids for 33 nm particle-size in the ambient condition was derived in ref.^13^ as follows:

13 $$\frac{{\mu }_{nf}}{{\mu }_{f}}=\mathrm{1/}(1-34.87{({d}_{p}/{d}_{f})}^{-0.3}{\phi }^{1.03})\mathrm{.}$$

The thermal conductivity ratio of water-Al_2_O_3_ nanofluids is calculated by the Corcione model^13^ is:

14 $$\frac{{k}_{nf}}{{k}_{f}}=1+4.4{{\rm{Re}}}_{B}^{0.4}{{\rm{\Pr }}}^{0.66}{(\frac{T}{{T}_{fr}})}^{10}{(\frac{{k}_{p}}{{k}_{f}})}^{0.03}{\phi }^{0.66}\mathrm{.}$$

where Re_*B*_ is the brownian motion Reynolds number which is defined as:

15 $${{\rm{Re}}}_{B}=\frac{{\rho }_{f}{u}_{B}{d}_{p}}{{\mu }_{f}}\mathrm{.}$$

and *u*_*B*_ is the brownian velocity of the nanoparticle which is calculated as:

16 $${u}_{B}=\frac{2{k}_{b}T}{\pi {\mu }_{f}{d}_{p}^{2}}.$$

where *k*_*b*_ = 1.380648 × 10^−23^ (*J*/*K*) is the Boltzmann constant. *l*_*f*_ = 0.17 nm is the mean path of fluid particles. *d*_*f*_ is the molecular diameter of water given as^13^

17 $${d}_{f}=\frac{6M}{N\pi {\rho }_{f}}.$$

where *M* is the molecular weight of the base fluid, *N* is the Avogadro number and *ρ*_*f*_ is the density of the base fluid at standard temperature (310 K). Accordingly, and basing on water as a base fluid, the value of *d*_*f*_ is obtained:

18 $${d}_{f}={(\frac{6\times 0.01801528}{6.022\times {10}^{23}\times \pi \times 998.26})}^{\mathrm{1/3}}=3.85\times {10}^{-10}\,{\rm{m}}\mathrm{.}$$

The electrical conductivity ratio $\frac{{\sigma }_{nf}}{{\sigma }_{f}}$ is defined by^43^

19 $$\frac{{\sigma }_{nf}}{{\sigma }_{f}}=1+\frac{3(\frac{{\sigma }_{p}}{{\sigma }_{f}}-1)\phi }{(\frac{{\sigma }_{p}}{{\sigma }_{f}}+2)-(\frac{{\sigma }_{p}}{{\sigma }_{f}}-1)\phi }.$$

Now we introduce the following non-dimensional variables:

20 $$\begin{array}{rcl}X & = & \frac{x}{L},\,Y=\frac{y}{L},\,{\bf{V}}=\frac{{\bf{v}}L}{{\nu }_{f}},\,P=\frac{p{L}^{2}}{{\rho }_{nf}{\nu }_{f}^{2}},\,{\phi }^{\ast }=\frac{\phi }{\varphi },\,{D}_{B}^{\ast }=\frac{{D}_{B}}{{D}_{B0}},\,{D}_{T}^{\ast }=\frac{{D}_{T}}{{D}_{T0}},\\ \delta  & = & \frac{{T}_{c}}{{T}_{h}-{T}_{c}},\,{\theta }_{nf}=\frac{{T}_{nf}-{T}_{c}}{{T}_{h}-{T}_{c}},\,{\theta }_{w}=\frac{{T}_{w}-{T}_{c}}{{T}_{h}-{T}_{c}},\,D=\frac{d}{L}\mathrm{.}\end{array}$$

Using the above variables yields the following dimensionless governing equations:

21 $$\nabla \cdot {\bf{V}}=\mathrm{0,}$$

22 $${\bf{V}}\cdot \nabla {\bf{V}}=-\,\nabla P+\frac{{\rho }_{f}}{{\rho }_{nf}}\frac{{\mu }_{nf}}{{\mu }_{f}}{\nabla }^{2}{\bf{V}}+\frac{{(\rho \beta )}_{nf}}{{\rho }_{nf}{\beta }_{f}}\frac{1}{{\rm{\Pr }}}Ra\cdot {\theta }_{nf}+\frac{{\rho }_{f}}{{\rho }_{nf}}\frac{{\sigma }_{nf}}{{\sigma }_{f}}{\bf{V}}\times {{\bf{B}}}^{\ast },$$

23 $$\begin{array}{rcl}{\bf{V}}\cdot \nabla {\theta }_{nf} & = & \frac{{(\rho {C}_{p})}_{f}}{{(\rho {C}_{p})}_{nf}}\frac{{k}_{nf}}{{k}_{f}}\frac{1}{{\rm{\Pr }}}{\nabla }^{2}{\theta }_{nf}+\frac{{(\rho {C}_{p})}_{f}}{{(\rho {C}_{p})}_{nf}}\frac{{D}_{B}^{\ast }}{{\rm{\Pr }}\cdot Le}\nabla {\phi }^{\ast }\cdot \nabla {\theta }_{nf}\\  &  & +\,\frac{{(\rho {C}_{p})}_{f}}{{(\rho {C}_{p})}_{nf}}\frac{{D}_{T}^{\ast }}{Pr\cdot Le\cdot {N}_{BT}}\frac{\nabla {\theta }_{nf}\cdot \nabla {\theta }_{nf}}{1+\delta {\theta }_{nf}},\end{array}$$

24 $${\bf{V}}\cdot \nabla {\phi }^{\ast }=\frac{{D}_{B}^{\ast }}{Sc}{\nabla }^{2}{\phi }^{\ast }+\frac{{D}_{T}^{\ast }}{Sc\cdot {N}_{BT}}\cdot \frac{{\nabla }^{2}{\theta }_{nf}}{1+\delta {\theta }_{nf}},$$

25 $${\nabla }^{2}{\theta }_{w}=0,$$

where **V** is the dimensionless velocity vector (*U*, *V*), B* is the dimensionless magnetic vector (*Ha*^2^ sin *γ*, *Ha*^2^ cos *γ*), ${D}_{B0}=\frac{{k}_{b}{T}_{c}}{3\pi {\mu }_{f}{d}_{p}}$ is the reference Brownian diffusion coefficient, ${D}_{T0}=0.26\frac{{k}_{f}}{2{k}_{f}+{k}_{p}}\frac{{\mu }_{f}}{{\rho }_{f}\theta }\varphi$ is the reference thermophoretic diffusion coefficient, *Sc* = *ν*_*f*_/*D*_*B*0_ is Schmidt number, *N*_*BT*_ = *ϕD*_*B*0_*T*_*c*_/*D*_*T*0_(*T*_*h*_ − *T*_*c*_) is the diffusivity ratio parameter (Brownian diffusivity/thermophoretic diffusivity), *Le* = *k*_*f*_*/*(*ρC*_*p*_)_*f*_*ϕD*_*B*0_ is Lewis number, *Ra* = *gρ*_*f*_*β*_*f*_(*T*_*h*_ − *T*_*c*_)*L*^3^*/*(*μ*_*f*_*α*_*f*_) is the Rayleigh number, $Ha={\bf{B}}L\sqrt{\frac{{\sigma }_{f}}{{\mu }_{f}}}$ is the Hartman number and *Pr* = *ν*_*f*_/*α*_*f*_ is the Prandtl number. The dimensionless boundary conditions of Eqs (25) and (36) are:

26 $$\begin{array}{rcl}U=V & = & 0,\frac{\partial {\phi }^{\ast }}{\partial n}=-\,\frac{{D}_{T}^{\ast }}{{D}_{B}^{\ast }}\cdot \frac{1}{{N}_{BT}}\cdot \frac{1}{1+\delta {\theta }_{nf}}\frac{\partial {\theta }_{nf}}{\partial n},\,{\theta }_{nf}=1\,{\rm{on}}\,\\  &  & 0.25\le X\le 0.75,Y=0,\end{array}$$

27 $$\begin{array}{rcl}U=V & = & 0,\frac{\partial {\phi }^{\ast }}{\partial n}=-\frac{{D}_{T}^{\ast }}{{D}_{B}^{\ast }}\cdot \frac{1}{{N}_{BT}}\cdot \frac{1}{1+\delta {\theta }_{nf}}\frac{\partial {\theta }_{nf}}{\partial n},\,{\theta }_{nf}=0\,{\rm{on}}\,\\  &  & 0\le X\le 0.25\,{\rm{and}}\,0.75\le X\le 1,\,Y=0.\end{array}$$

28 $$\begin{array}{rcl}U=V & = & 0,\frac{\partial {\phi }^{\ast }}{\partial n}=-\,\frac{{D}_{T}^{\ast }}{{D}_{B}^{\ast }}\cdot \frac{1}{{N}_{BT}}\cdot \frac{1}{1+\delta {\theta }_{nf}}\frac{\partial {\theta }_{nf}}{\partial n},\,{\theta }_{nf}=1\,{\rm{on}}\,\\  &  & 0.375\le Y\le 0.625,\,X=0,X=1,\end{array}$$

29 $$\begin{array}{rcl}U=V & = & 0,\frac{\partial {\phi }^{\ast }}{\partial n}=-\,\frac{{D}_{T}^{\ast }}{{D}_{B}^{\ast }}\cdot \frac{1}{{N}_{BT}}\cdot \frac{1}{1+\delta {\theta }_{nf}}\frac{\partial {\theta }_{nf}}{\partial n},{\theta }_{nf}=0\,{\rm{on}}\,\\  &  & 0\le Y\le 0.375\,{\rm{and}}\,0.625\le Y\le 1,X=0,X=1.\end{array}$$

30 $$U=V=0,\,\frac{\partial {\phi }^{\ast }}{\partial n}=0,\,\frac{\partial {\theta }_{nf}}{\partial n}=0\,{\rm{on}}\,0\le X\le 1Y=1.$$

31 $${\theta }_{nf}={\theta }_{w},\,{\rm{at}}\,{\rm{the}}\,{\rm{outer}}\,{\rm{solid}}\,{\rm{square}}\,{\rm{surface}},$$

32 $$U=V=0,\frac{\partial {\phi }^{\ast }}{\partial n}=-\,\frac{{D}_{T}^{\ast }}{{D}_{B}^{\ast }}\cdot \frac{1}{{N}_{BT}}\cdot \frac{1}{1+\delta {\theta }_{nf}}\frac{\partial {\theta }_{nf}}{\partial n},\frac{\partial {\theta }_{nf}}{\partial n}={K}_{r}\frac{\partial {\theta }_{w}}{\partial n},X,Y\,{\rm{in}}\,[\frac{(1-D)}{2},\frac{(1+D)}{2}].$$

where *K*_*r*_ = *k*_*w*_/*k*_*nf*_ is the thermal conductivity ratio and *D* = *d*/*L* is the aspect ratio of inner square cylinder width to the outer square cylinder width.

The local Nusselt number evaluated at the left and bottom walls, which is defined by

33 $$N{u}_{l}=-\,\frac{{k}_{nf}}{{k}_{f}}{(\frac{\partial {\theta }_{nf}}{\partial X})}_{X=0},\,N{u}_{b}=-\frac{{k}_{nf}}{{k}_{f}}{(\frac{\partial {\theta }_{nf}}{\partial Y})}_{Y=0},$$

Finally, the average Nusselt numbers evaluated at the heated parts of the left, right and bottom walls of the square cavity which are given respectively by:

34 $${\overline{Nu}}_{l}={\int }_{0.375}^{0.625}N{u}_{l}{\rm{d}}Y,\,{\overline{Nu}}_{r}={\int }_{0.375}^{0.625}N{u}_{r}{\rm{d}}Y,\,{\overline{Nu}}_{b}={\int }_{0.25}^{0.75}N{u}_{b}{\rm{d}}X,$$

and

35 $${\overline{Nu}}_{nf}={\overline{Nu}}_{l}+{\overline{Nu}}_{r}+{\overline{Nu}}_{b}.$$

where $$N{u}_{r}=-\,\frac{{k}_{nf}}{{k}_{f}}{(\frac{\partial {\theta }_{nf}}{\partial X})}_{X=1},$$

## Numerical Method and Validation

An iterative finite difference method (FDM) is employed to solve the governing Equations (25–36) subject to the boundary conditions (26–32).

Continuity equation and momentum equation:

36 $$\nabla \cdot {\bf{V}}=0,$$

37 $${\bf{V}}\cdot \nabla V=-\,\nabla P+\frac{{\rho }_{f}}{{\rho }_{nf}}\frac{{\mu }_{nf}}{{\mu }_{f}}{\nabla }^{2}{\bf{V}}+\frac{{(\rho \beta )}_{nf}}{{\rho }_{nf}{\beta }_{f}}\frac{1}{{\rm{\Pr }}}Ra{\theta }_{nf}+\frac{{\rho }_{f}}{{\rho }_{nf}}\frac{{\sigma }_{nf}}{{\sigma }_{f}}{\bf{V}}\times {{\bf{B}}}^{\ast },$$

Expanding the equations then yields

Continuity equation:

38 $$\frac{\partial U}{\partial X}+\frac{\partial V}{\partial Y}=0$$

Momentum equation in the *X*-direction:

39 $$\begin{array}{rcl}(\frac{{\rho }_{nf}}{{\rho }_{f}})U\frac{\partial U}{\partial X}+(\frac{{\rho }_{nf}}{{\rho }_{f}})V\frac{\partial U}{\partial Y} & = & -(\frac{{\rho }_{nf}}{{\rho }_{f}})\frac{\partial P}{\partial X}+(\frac{{\mu }_{nf}}{{\mu }_{f}})(\frac{{\partial }^{2}U}{\partial {X}^{2}}+\frac{{\partial }^{2}U}{\partial {Y}^{2}})\\  &  & +\,\frac{{\rho }_{f}}{{\rho }_{nf}}\frac{{\sigma }_{nf}}{{\sigma }_{f}}H{a}^{2}(V\,\sin \,\gamma \,\cos \,\gamma -U\,{\sin }^{2}\gamma )\end{array}$$

Momentum equation in the *Y*-direction:

40 $$\begin{array}{rcl}(\frac{{\rho }_{nf}}{{\rho }_{f}})U\frac{\partial V}{\partial X}+(\frac{{\rho }_{nf}}{{\rho }_{f}})V\frac{\partial V}{\partial Y} & = & -(\frac{{\rho }_{nf}}{{\rho }_{f}})\frac{\partial P}{\partial Y}+(\frac{{\mu }_{nf}}{{\mu }_{f}})(\frac{{\partial }^{2}V}{\partial {X}^{2}}+\frac{{\partial }^{2}V}{\partial {Y}^{2}})\\  &  & +\,[(\frac{{(\rho \beta )}_{nf}}{{(\rho \beta )}_{f}})\frac{1}{Pr}Ra\theta ]+\frac{{\rho }_{f}}{{\rho }_{nf}}\frac{{\sigma }_{nf}}{{\sigma }_{f}}H{a}^{2}(U\,\sin \,\gamma \,\cos \,\gamma -V\,{\cos }^{2}\gamma )\end{array}$$

Now we introduce the stream function and vorticity:

41 $$U=\frac{\partial {\rm{\Psi }}}{\partial Y},\,V=-\,\frac{\partial {\rm{\Psi }}}{\partial X}$$

42 $${\rm{\Omega }}=(\frac{\partial V}{\partial X}-\frac{\partial U}{\partial Y})$$

The stream function defined above automatically satisfies the continuity equation. The vorticity equation is obtained by eliminating the pressure between the two momentum equations, i.e. by taking the *Y*-derivative of the *X*-momentum and subtracting from it the *X*-derivative of the *Y*-momentum:

43 $$\begin{array}{l}(\frac{{\rho }_{nf}}{{\rho }_{f}})[\frac{\partial U}{\partial Y}\frac{\partial U}{\partial X}+U\frac{{\partial }^{2}U}{\partial Y\partial X}+\frac{\partial V}{\partial Y}\frac{\partial U}{\partial Y}+V\frac{{\partial }^{2}U}{\partial {Y}^{2}}-\frac{\partial U}{\partial x}\frac{\partial V}{\partial X}-U\frac{{\partial }^{2}V}{\partial {X}^{2}}-\frac{\partial V}{\partial X}\frac{\partial V}{\partial Y}\\ -V\frac{{\partial }^{2}V}{\partial X\partial Y}]=(\frac{{\mu }_{nf}}{{\mu }_{f}})(\frac{{\partial }^{3}U}{\partial Y\partial {X}^{2}}+\frac{{\partial }^{3}U}{\partial {Y}^{3}}-\frac{{\partial }^{3}V}{\partial {X}^{3}}-\frac{{\partial }^{3}V}{\partial X\partial {Y}^{2}})-[(\frac{{(\rho \beta )}_{nf}}{{(\rho \beta )}_{f}})\frac{1}{{\rm{\Pr }}}Ra\frac{\partial \theta }{\partial X}]\\ +\frac{{\rho }_{f}}{{\rho }_{nf}}\frac{{\sigma }_{nf}}{{\sigma }_{f}}H{a}^{2}(\frac{\partial U}{\partial X}\,\sin \,\gamma \,\cos \,\gamma -\frac{\partial V}{\partial X}\,{\cos }^{2}\gamma -\frac{\partial V}{\partial Y}\,\sin \,\gamma \,\cos \,\gamma +\frac{\partial U}{\partial Y}\,{\sin }^{2}\gamma )\end{array}$$

which simplifies to:

44 $$\begin{array}{l}(\frac{{\rho }_{nf}}{{\rho }_{f}})[U(\frac{{\partial }^{2}V}{\partial {X}^{2}}-\frac{{\partial }^{2}U}{\partial Y\partial X})+V(\frac{{\partial }^{2}V}{\partial X\partial Y}-\frac{{\partial }^{2}U}{\partial {Y}^{2}})+\frac{\partial V}{\partial X}(\frac{\partial U}{\partial X}+\frac{\partial V}{\partial Y})-\frac{\partial U}{\partial Y}(\frac{\partial U}{\partial X}+\frac{\partial V}{\partial Y})]\\ =(\frac{{\mu }_{nf}}{{\mu }_{f}})[(\frac{{\partial }^{3}V}{\partial {X}^{3}}-\frac{{\partial }^{3}U}{\partial Y\partial {X}^{2}})+(\frac{{\partial }^{3}V}{\partial X\partial {Y}^{2}}-\frac{{\partial }^{3}U}{\partial {Y}^{3}})]-[(\frac{{(\rho \beta )}_{nf}}{{(\rho \beta )}_{f}})\frac{1}{\Pr }Ra\frac{\partial \theta }{\partial x}]\\ \,\,\,+\frac{{\rho }_{f}}{{\rho }_{nf}}\frac{{\sigma }_{nf}}{{\sigma }_{f}}H{a}^{2}(\frac{\partial U}{\partial Y}{\sin }^{2}\gamma +\frac{\partial U}{\partial X}\,\sin \,\gamma \,\cos \,\gamma -\frac{\partial V}{\partial Y}\,\sin \,\gamma \,\cos \,\gamma -\frac{\partial V}{\partial X}{\cos }^{2}\gamma )\end{array}$$

Using the definition of stream function we obtain:

45 $$\begin{array}{rcl}(\frac{{\mu }_{nf}}{{\mu }_{f}})[\frac{{\partial }^{2}{\rm{\Omega }}}{\partial {X}^{2}}+\frac{{\partial }^{2}{\rm{\Omega }}}{\partial {Y}^{2}}] & = & (\frac{{\rho }_{nf}}{{\rho }_{f}})(\frac{\partial {\rm{\Psi }}}{\partial Y}\frac{\partial \omega }{\partial X}-\frac{\partial {\rm{\Psi }}}{\partial X}\frac{\partial \omega }{\partial Y})+[(\frac{{(\rho \beta )}_{nf}}{{(\rho \beta )}_{f}})\frac{1}{{\rm{\Pr }}}Ra\frac{\partial \theta }{\partial X}]\\  &  & +\,\frac{{\rho }_{f}}{{\rho }_{nf}}\frac{{\sigma }_{nf}}{{\sigma }_{f}}H{a}^{2}(\frac{{\partial }^{2}{\rm{\Psi }}}{\partial {Y}^{2}}{\sin }^{2}\gamma +2\frac{{\partial }^{2}{\rm{\Psi }}}{\partial X\partial Y}\,\sin \,\gamma \,\cos \,\gamma +\frac{{\partial }^{2}{\rm{\Psi }}}{\partial {X}^{2}}\,{\cos }^{2}\gamma )\end{array}$$

In terms of the stream function, the equation defining vorticity becomes:

46 $$(\frac{{\partial }^{2}{\rm{\Psi }}}{\partial {X}^{2}}+\frac{{\partial }^{2}{\rm{\Psi }}}{\partial {Y}^{2}})=-\,{\rm{\Omega }}$$

Since the energy, volume fraction equation and block conduction do not contain the pressure variable then the velocity in these equations are easily transformed into stream function formulation.

The finite difference form of equation relating the dimensionless vorticity is:

47 $$(\frac{{\mu }_{nf}}{{\mu }_{f}})[\frac{{{\rm{\Omega }}}_{i+1,j}-2{{\rm{\Omega }}}_{i,j}+{{\rm{\Omega }}}_{i-1,j}}{{({\rm{\Delta }}X)}^{2}}+\frac{{{\rm{\Omega }}}_{i,j+1}-2{{\rm{\Omega }}}_{i,j}+{{\rm{\Omega }}}_{i,j-1}}{{({\rm{\Delta }}Y)}^{2}}]-{({S}_{{\rm{\Omega }}})}_{i,j}=0$$

with

48 $$\begin{array}{rcl}{({S}_{{\rm{\Omega }}})}_{i,j} & = & (\frac{{\rho }_{nf}}{{\rho }_{f}})[(\frac{{{\rm{\Psi }}}_{i,j+1}-{{\rm{\Psi }}}_{i,j-1}}{2{\rm{\Delta }}Y})(\frac{{{\rm{\Omega }}}_{i+\mathrm{1,}j}-{{\rm{\Omega }}}_{i-\mathrm{1,}j}}{2{\rm{\Delta }}X})-(\frac{{{\rm{\Psi }}}_{i+\mathrm{1,}j}-{{\rm{\Psi }}}_{i-\mathrm{1,}j}}{2{\rm{\Delta }}X})(\frac{{{\rm{\Omega }}}_{i,j+1}-{{\rm{\Omega }}}_{i,j-1}}{2{\rm{\Delta }}Y})]\\  &  & +\,(\frac{{(\rho \beta )}_{nf}}{{(\rho \beta )}_{f}})\frac{1}{Pr}Ra(\frac{{\theta }_{i+\mathrm{1,}j}-{\theta }_{i-\mathrm{1,}j}}{2{\rm{\Delta }}X})+\frac{{\rho }_{f}}{{\rho }_{nf}}\frac{{\sigma }_{nf}}{{\sigma }_{f}}H{a}^{2}(\frac{{{\rm{\Psi }}}_{i,j+1}-2{{\rm{\Psi }}}_{i,j}+{{\rm{\Psi }}}_{i,j-1}}{{({\rm{\Delta }}Y)}^{2}}\,{\sin }^{2}\gamma \\  &  & +\,\frac{{{\rm{\Psi }}}_{i+\mathrm{1,}j+1}-{{\rm{\Psi }}}_{i+\mathrm{1,}j}-{{\rm{\Psi }}}_{i-\mathrm{1,}j-1}+{{\rm{\Psi }}}_{i-\mathrm{1,}j}}{2{\rm{\Delta }}X{\rm{\Delta }}Y}\,\sin \,\gamma \,\cos \,\gamma \,+\frac{{{\rm{\Psi }}}_{i+\mathrm{1,}j}-2{{\rm{\Psi }}}_{i,j}+{{\rm{\Psi }}}_{i-\mathrm{1,}j}}{{({\rm{\Delta }}X)}^{2}}\,{\cos }^{2}\gamma )\end{array}$$

In order to solve for the value of Ω at the grid point *i*, *j*, the values of Ω at the right–hand side must be provided. *B* = Δ*X*/Δ*Y*. This method is known as the point Gauss–Seidel method. The general formulation of the method provides:

49 $$\begin{array}{rcl}{{\rm{\Omega }}}_{i,j}^{k+1} & = & {{\rm{\Omega }}}_{i,j}^{k}+\frac{{\lambda }_{r}}{2(1+{B}^{2})}[{{\rm{\Omega }}}_{i+1,j}^{k}+{{\rm{\Omega }}}_{i-1,j}^{k+1}+{B}^{2}({{\rm{\Omega }}}_{i,j+1}^{k}+{{\rm{\Omega }}}_{i,j-1}^{k+1})\\  &  & -2(1+{B}^{2}){{\rm{\Omega }}}_{i,j}^{k}-(\frac{{\mu }_{f}}{{\mu }_{nf}}){({\rm{\Delta }}X)}^{2}{({S}_{{\rm{\Omega }}})}_{i,j}^{k}]\end{array}$$

The computation is assumed to move through the grid points from left to right and bottom to top. Here, the superscript *k* denotes the iteration number. We make partition the solution domain in the *X* − *Y* plane into equal rectangles of sides Δ*X* and Δ*Y*. The values of the relaxation parameter *λ*_*r*_ must lie in the range 0 < *λ*_*r*_ < 2 for convergence. The range 0 < *λ*_*r*_ < 1 corresponds to under–relaxation, 1 < *λ*_*r*_ < 2 over–relaxation and *λ*_*r*_ = 1 refers to the Gauss–Seidel iteration. The finite difference form of equation relating the stream function, energy and volume fraction could be treat in the same way.

The grid–points distribution at the adiabatic inner block and the square cavity is shown in Fig. 1(b), where *ND* is number of node points in the horizontal and vertical axis of the adiabatic inner block. The temperature conditions at the left and bottom interfaces are:

50 $$\begin{array}{rcl}{({\theta }_{nf})}_{\frac{NX}{2}-ND+1,j}^{k+1} & = & {({\theta }_{w})}_{\frac{NX}{2}-ND+1,j}^{k}\\ {({\theta }_{w})}_{\frac{NX}{2}-ND+1,j}^{k+1} & = & [(\frac{1}{Kr})(-{({\theta }_{nf})}_{\frac{NX}{2}-ND-1,j}^{k}+4{({\theta }_{nf})}_{\frac{NX}{2}-ND,j}^{k}-3{({\theta }_{nf})}_{\frac{NX}{2}-ND+1,j}^{k+1})\\  &  & +\,4{({\theta }_{w})}_{\frac{NX}{2}-ND+2,j}^{k}-{({\theta }_{w})}_{\frac{NX}{2}-ND+3,j}^{k}]\,/3\end{array}$$

51 $$\begin{array}{rcl}{({\theta }_{nf})}_{i,\frac{NY}{2}-ND+1}^{k+1} & = & {({\theta }_{w})}_{i,\frac{NY}{2}-ND+1}^{k}\\ {({\theta }_{w})}_{i,\frac{NY}{2}-ND+1}^{k+1} & = & [(\frac{1}{{K}_{r}})(-{({\theta }_{nf})}_{i,\frac{NY}{2}-ND-1}^{k}+4{({\theta }_{nf})}_{i,\frac{NY}{2}-ND}^{k}-3{({\theta }_{nf})}_{i,\frac{NY}{2}-ND+1}^{k+1})\\  &  & +4{({\theta }_{w})}_{i,\frac{NY}{2}-ND+2}^{k}-{({\theta }_{w})}_{i,\frac{NY}{2}-ND+3}^{k}]\,/3\end{array}$$

**Figure 2:**
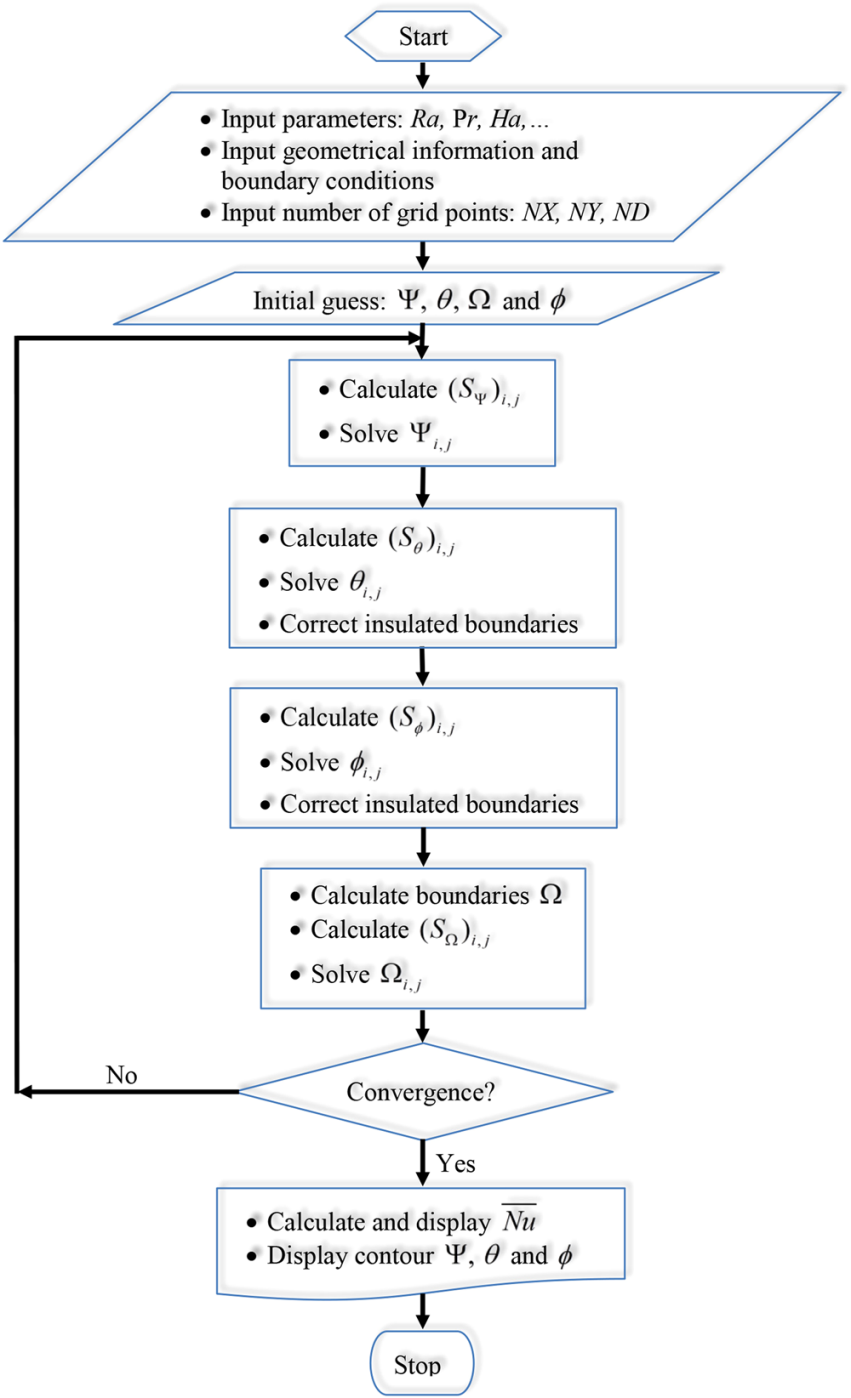
Flowchart for the solution procedure of MHD convective heat transfer in a square cavity with conductive inner block.

The convergence of the solution is assumed when the relative error for each of the variables satisfies the following convergence criterium:

$$|\frac{{{\rm{\Gamma }}}^{i+1}-{{\rm{\Gamma }}}^{i}}{{{\rm{\Gamma }}}^{i+1}}|\le \eta ,$$

where *i* represents the iteration number and *η* is the convergence criterion. In this study, the convergence criterion was set at *η* = 10^−5^. The flowchart of the solution method of MHD convective heat transfer in a square cavity with conductive inner block is presented in Fig. 2.

In the present paper, several grid testings are performed: 10 × 10, 20 × 20, 40 × 40, 60 × 60, 80 × 80, 100 × 100, 120 × 120, 140 × 140 and 160 × 160. Table 1 shows the calculated strength of the flow circulation (Ψ_min_) and average Nusselt number $({\overline{Nu}}_{nf})$ at different grid sizes for *Ra* = 10^5^, *Ha* = 15, *ϕ* = 0.02, *k*_*w*_ = 0.76 and *D* = 0.3. The results show insignificant differences for the 140 × 140 grids and above. Therefore, for all computations in this paper for similar problems to this subsection, the 140 × 140 uniform grid is employed.

**Table 1:** Grid testing for Ψ_min_, Ψ_max_ and ${\overline{Nu}}_{nf}$ at different grid size for *Ra* = 10^5^, *Ha* = 15, *ϕ* = 0.02, *k*_*w*_ = 0.76 and *D* = 0.3.

Grid size	Ψ_min_	Ψ_max_	${\overline{Nu}}_{nf}$
10 × 10	−0.8531	0.71976	4.6902
20 × 20	−0.87489	0.72082	4.9823
40 × 40	−0.89804	0.72647	5.2859
60 × 60	−0.91317	0.73159	5.4331
80 × 80	−0.93065	0.73535	5.598
100 × 100	−0.95656	0.73813	5.6167
120 × 120	−0.96098	0.73988	5.6255
140 × 140	−0.96099	0.74058	5.6261
160 × 160	−0.96156	0.74067	5.6265

**Figure 3:**
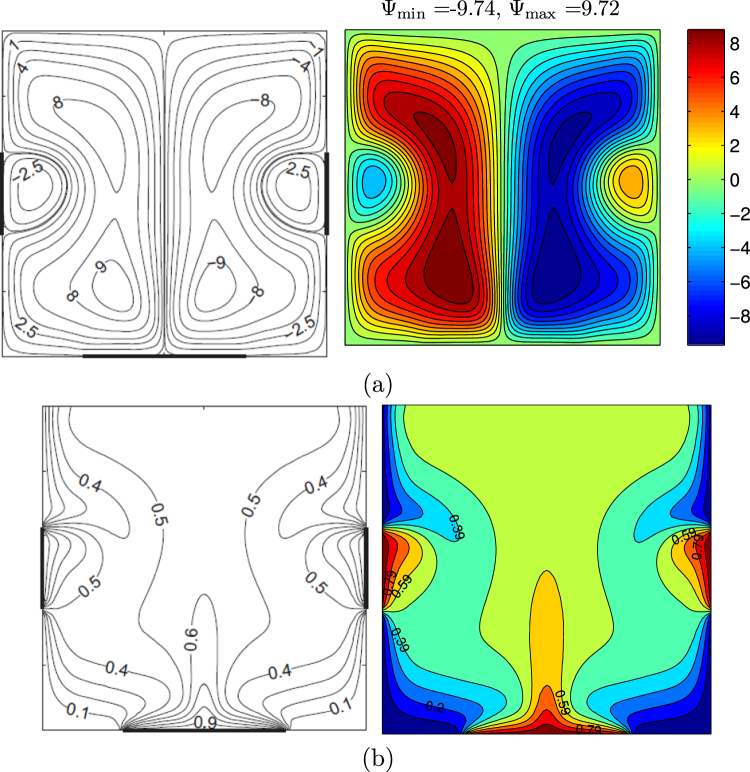
Streamlines (**a**)^4^, (left), present study (right), isotherms (**b**)^4^, (left), present study (right) *Ra* = 10^6^, *ϕ* = 0 and *D* = 0.5.

**Figure 4:**
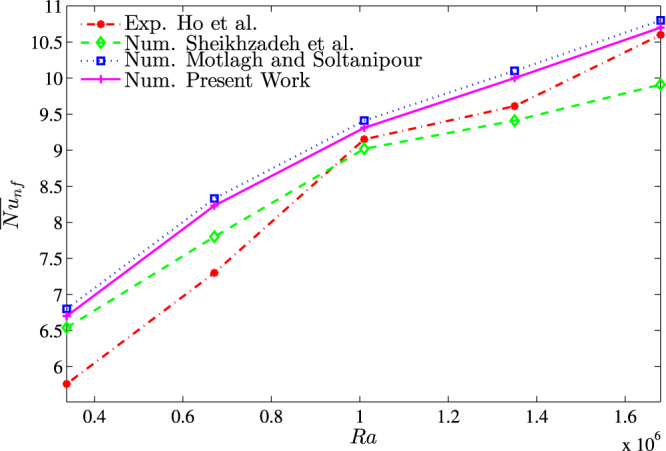
Comparison of the mean Nusselt number obtained from present numerical simulation with the experimental results of Ho *et al*.^44^, numerical results of Sheikhzadeh *et al*.^45^ and numerical results of Motlagh and Soltanipour^20^ for different values of Rayleigh numbers.

**Figure 5:**
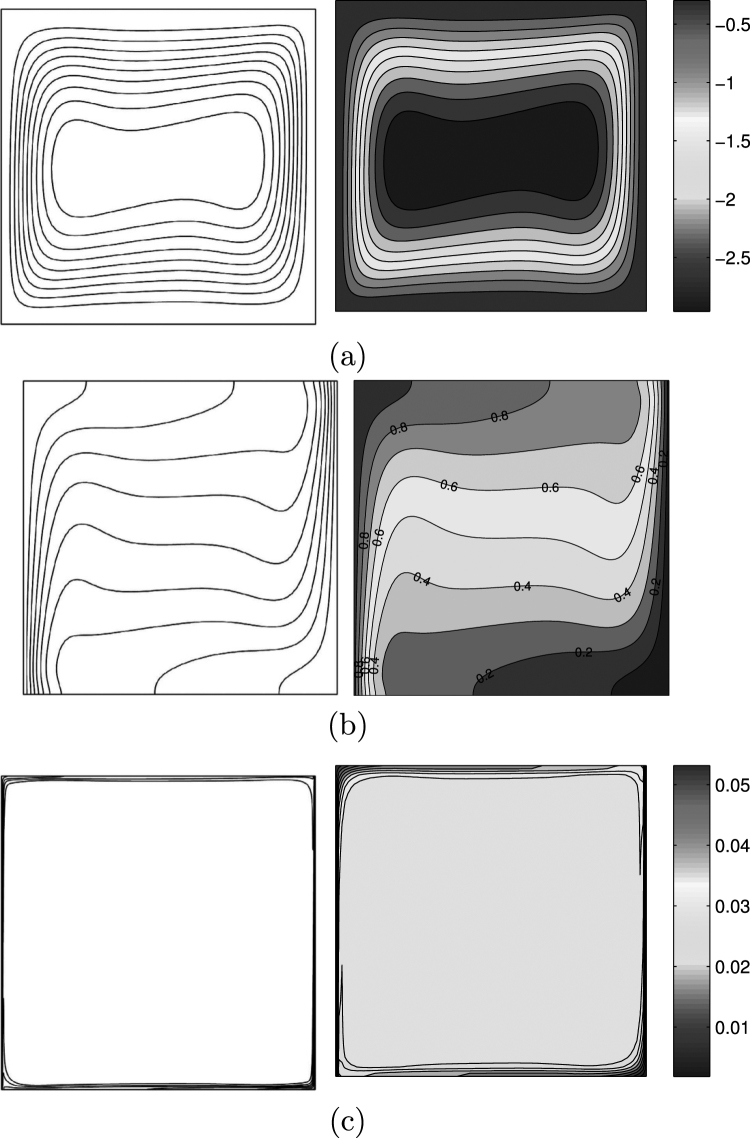
Corcione *et al*.^46^ (left), present study (right) for streamlines (**a**) Isotherms (**b**) and nanoparticle distribution **(c**) at *Ra* = 3.37×10^5^, *ϕ* = 0.04 and *D* = 0.

**Figure 6:**
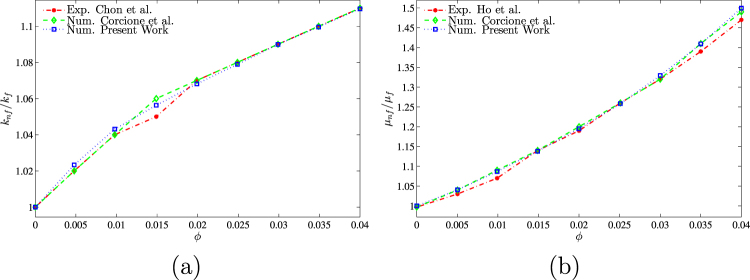
Comparison of (**a**) thermal conductivity ratio with Chon *et al*.^49^ and Corcione *et al*.^46^ and (**b**) dynamic viscosity ratio with Ho *et al*.^44^ and Corcione *et al*.^46^.

For the validation of data, the results are compared with previously published numerical results obtained by Kaluri and Basak^4^ for the case of natural convection heat transfer in discretely heated porous square cavity, as shown in Fig. 3. In addition, a comparison of the average Nusselt number is made between the resulting figure and the experimental results provided by Ho *et al*.^44^ and the numerical results provided by Sheikhzadeh *et al*.^45^ and by Motlagh and Soltanipour^20^ for the case of the natural convection of Al_2_O_3_-water nanofluid in a square cavity using Buongiorno’s two-phase model as shown in Fig. 4. Next, a comparisons made between the present streamlines, isotherms, nanoparticles volume fraction and the average Nusselt number results and the numerical one obtained by Corcione *et al*.^46^ are demonstrated in Fig. 5. Figure 6 presents alternative comparisons regarding the enhancement in the thermal conductivity due to the addition of the Al_2_O_3_ nanoparticles with two different experimental results and the numerical results of Corcione *et al*.^46^ as well. These results provide confidence to the accuracy of the present numerical method.

## Results and Discussion

In this section, we present numerical results for the streamlines, isotherms and nanoparticle distribution with various values of the nanoparticle volume fraction (0 ≤ *ϕ* ≤ 0.04), the Rayleigh number (10^2^ ≤ *Ra* ≤ 10^6^), Hartmann number (0 ≤ *Ha* ≤ 50), thermal conductivity of the conductive inner block (*k*_*w*_ = 0.28, 0.76, 1.95, 7 and 16) (epoxy: 0.28, brickwork: 0.76, granite: 1.95, solid rock: 7, stainless steel: 16), length of the conductive inner block (0 ≤ *D* ≤ 0.7), where the values of Prandtl number, Lewis number, Schmidt number, inclination angle of magnetic field, ratio of Brownian to thermophoretic diffusivity and normalized temperature parameter are fixed at Pr = 4.623, *Le* = 3.5 × 10^5^, *Sc* = 3.55 × 10^4^, $\gamma =\frac{\pi }{4}$, *N*_*BT*_ = 1.1 and *δ* = 155. The values of the average Nusselt number are calculated for various values of *Ra*, *ϕ* and *D*. The thermophysical properties of the base fluid (water) and solid Al_2_O_3_ phases are tabulated in Table 2.

**Table 2:** Thermo-physical properties of water with Al_2_O_3_ nanoparticles at *T* = 310 K^20,48^.

Physical properties	Fluid phase (water)	Al_2_O_3_
*C*_*p*_ (J/kgK)	4178	765
*ρ* (kg/m^3^)	993	3970
*k* (Wm^−1^ K^−1^)	0.628	40
*β* × 10^5^ (1/K)	36.2	0.85
*μ* × 10^6^ (kg/ms)	695	—
*d*_*p*_ (nm)	0.385	33
*σ* (Sm^−1^)	0.05	1 × 10^−10^

The contour level legends define the direction of the fluid heat flow (clockwise or anti-clockwise direction) and also the strength of the flow. Positive values of Ψ denotes the anti-clockwise fluid heat flow, whereas negative designates the clockwise fluid heat flow. Ψ_min_ represents the extreme values of the stream function. These values are important to show the minimum change of the flow.

**Figure 7:**
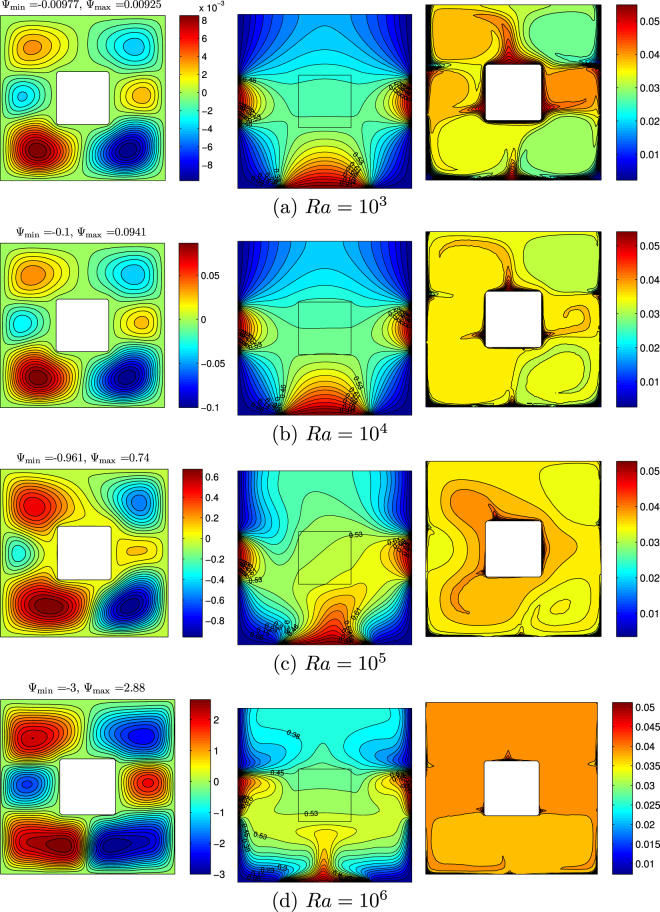
Variation of the streamlines (left), isotherms (middle), and nanoparticle distribution (right) evolution by Rayleigh number (*Ra*) for *ϕ* = 0.02, *Ha* = 15, *k*_*w*_ = 0.76 and *D* = 0.3.

Figure 7 presents streamlines, isotherms and nanoparticles isoconcentrations for different values of the Rayleigh number at *ϕ* = 0.02, *Ha* = 15, *k*_*w*_ = 0.76 and *D* = 0.3. In the case of low Rayleigh number values (Fig. 6a,b) one can find a formation of six convective cells inside the cavity, namely three vortexes for left and right sides from the centered solid block. More intensive circulations are located under the solid block where heater has an essential size in comparison with two others. In this part we have an ascending convective flow in central zone of the heater and descending ones near the cooled vertical walls. An appearance of two cells on the left (clockwise circulation) and right (counter-clockwise circulation) parts of the solid block can be explained by a formation of horizontal temperature difference between the vertical heaters and cold medium descended from the upper part of cavity where we have two vertical coolers. At the same time two convective cells are formed in the upper part of the cavity due to the effects of vertical coolers. In the case of low Rayleigh numbers the heat transfer regime is a heat conduction where isotherms are quasi-parallel to the isothermal zones. Non-homogeneous distributions of nanoparticles inside the cavity for low Rayleigh numbers are due to an essential effect of thermophoresis where we have the nanoparticles motion along the heat flux from heated zones to cooled ones. Moreover, the abovementioned circulations also characterize zones of nanoparticles motion. These zones illustrate distribution of nanoparticles of different concentrations. Moderate and high values of the Rayleigh number (Fig. 7c,d) reflect more intensive nanofluid circulation inside the cavity, where all six circulations become more intensive. In these cases convective heat transfer is dominated heat transfer regime. Therefore, it is possible to observe a development of asymmetry flow and heat transfer behavior (Fig. 7c) in the case of symmetry boundary conditions with respect to the vertical line *X* = 0.5. For these Ra values thermal plumes become stronger and thermal boundary layers thicknesses decrease. Distributions of nanoparticles for high Ra is more homogeneous due to non-essential effect of thermophoresis. The same effect has been described earlier by Sheremet *et al*.^47^.

**Figure 8:**
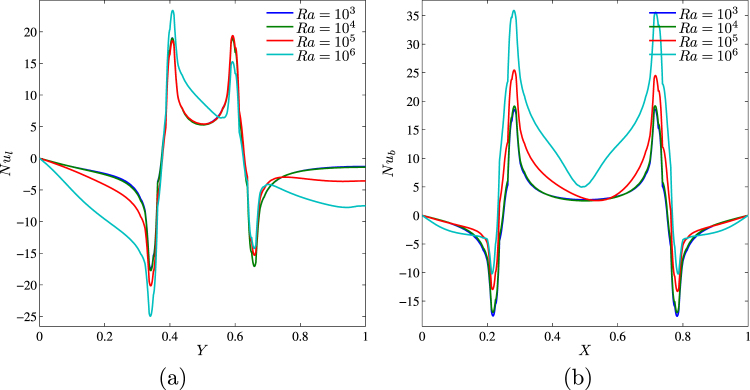
Variation of local Nusselt number interfaces with (**a**) *Y* and (**b**) *X* for different *Ra* at *ϕ* = 0.02, *Ha* = 15, *k*_*w*_ = 0.76 and *D* = 0.3.

Profiles of the local Nusselt number along the left and bottom walls are shown in Fig. 8. First of all, it is possible to conclude that local Nusselt number has the same values for *Ra* = 10^3^ and 10^4^ and these distributions are quasi-symmetry with respect to *Y* = 0.5 (Fig. 8a) and *X* = 0.5 (Fig. 8b) due to a domination of the heat conduction. Further growth of the Rayleigh number leads to an increase in the absolute value of the local Nusselt number and asymmetrical distribution due to more intensive convective flow and heat transfer regime. For the fixed value of *Ra* along the vertical wall (Fig. 8a), behavior of the local Nusselt number can be described as follows, an increase in *Y* from 0 to 0.375 reflects an augmentation of *Nu*_*l*_ due to an interaction between the hot fluid and cold bottom part of the vertical wall. Negative values of *Nu*_*l*_ in this part can be explained by the direction of heat flux from the fluid to the wall. For 0.375 < *Y* < 0.4 one can find an increase in *Nu*_*l*_ with successive reduction and growth of *Nu*_*l*_. Such changes occur near the bottom part of the heater due to a variation of the heat flux direction and heating of the nanofluid from the vertical wall. Further decrease and increase in *Nu*_*l*_ occur along the heater where high values of the heat transfer rate are near the bottom and upper ends of the heater and low value is in central part where fluid is hot and, as a result temperature difference is low. A growth of *Y* > 0.6 repeats the bottom part distribution of *Nu*_*l*_. Behavior of *Nu*_*b*_ (Fig. 8b) is the same like above-described for *Nu*_*l*_.

**Figure 9:**
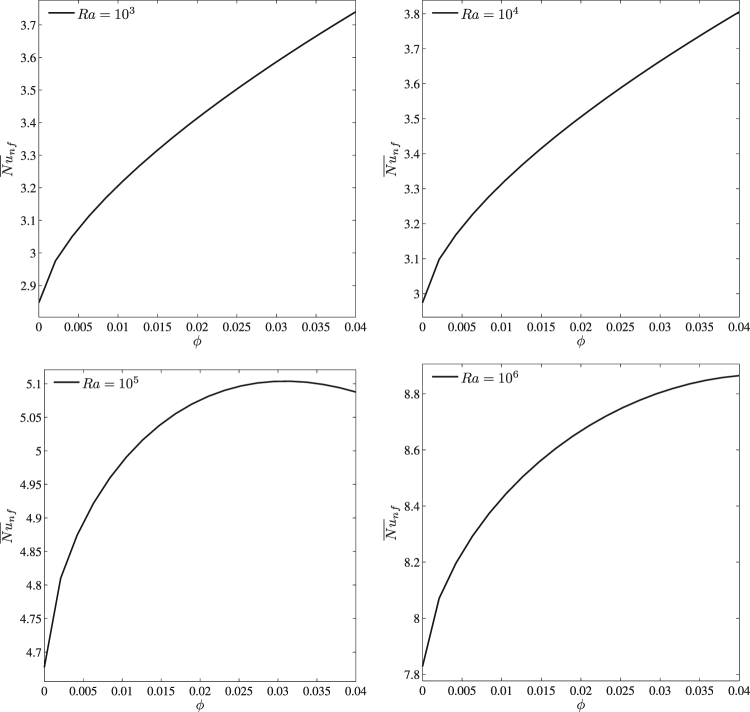
Variation of average Nusselt number with *ϕ* for different *Ra* at *Ha* = 0, *k*_*w*_ = 0.76 and *D* = 0.3.

Effects of the Rayleigh number and nanoparticles volume fraction on the average Nusselt number (see Eqs (33–35)) are shown in Fig. 9. As has been mentioned above, low Rayleigh numbers (10^3^ and 10^4^) illustrate a growth of the average Nusselt number with nanoparticles volume fraction, while for *Ra* = 10^5^ one can find a formation of maximum heat transfer rate for *ϕ* = 0.03. Such behavior can be explained by a formation of asymmetry convective flow and heat transfer regimes (see Fig. 7c). In the case of *Ra* = 10^6^ we have the heat transfer enhancement with *ϕ*.

**Figure 10:**
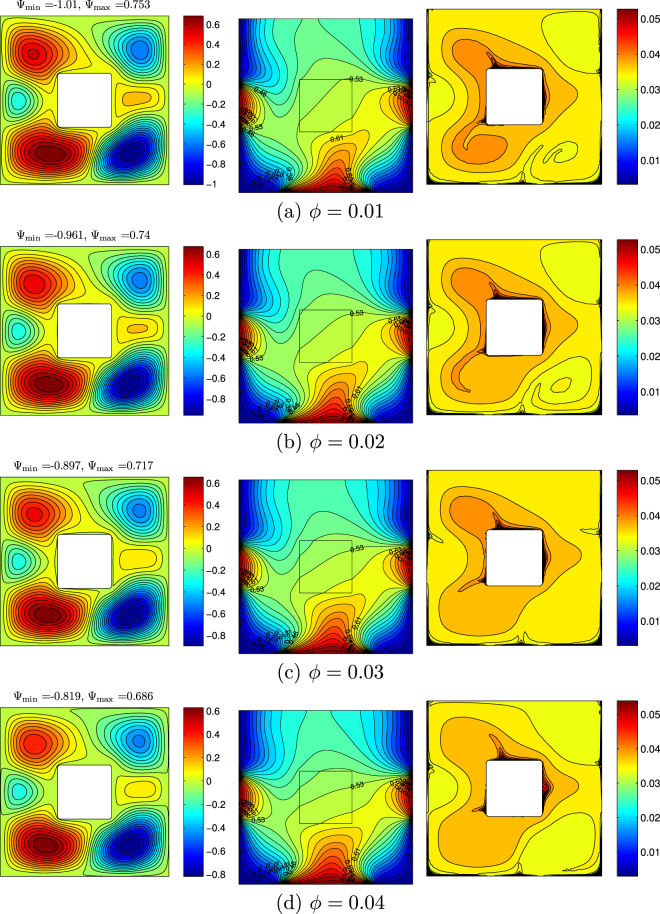
Variation of the streamlines (left), isotherms (middle), and nanoparticle distribution (right) evolution by nanoparticles volume fraction (*ϕ*) for *Ra* = 10^5^, *Ha* = 15, *K*_*w*_ = 0.76 and *D* = 0.3.

An influence of nanoparticles volume fraction on streamlines, isotherms and nanoparticles isoconcentrations is demonstrated in Fig. 10. It should be noted that distributions of stream function and temperature does not change with *ϕ*. One can find only a reduction of fluid flow rate with the nanoparticles volume fraction due to an increase in the effective viscosity (see Eq. (13)). Variation of nanoparticles isoconcentrations is more essential, namely, a growth of *ϕ* leads to more homogeneous distributions of nanoparticles inside the cavity.

**Figure 11:**
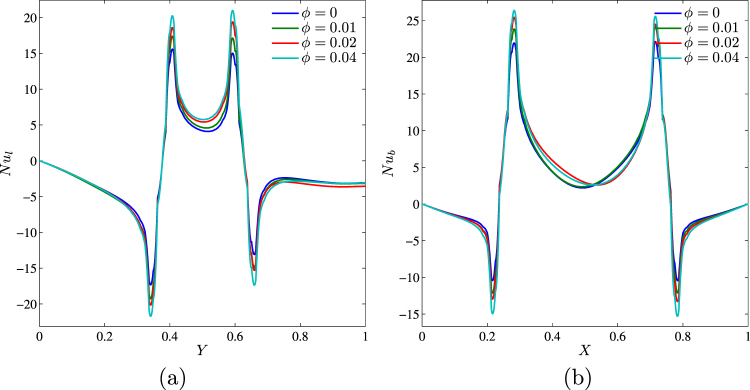
Variation of local Nusselt number interfaces with (**a**) *Y* and (**b**) *X* for different *ϕ* at *Ra* = 10^5^, *Ha* = 15, *k*_*w*_ = 0.76 and *D* = 0.3.

Variations of the local Nusselt number along the left vertical wall and bottom horizontal wall are presented in Fig. 11. Nature of the local Nusselt number has been described in detail above. It is worth noting here that an increase in *ϕ* leads to a growth of local Nusselt number, while behavior of *Nu*_*l*_ and *Nu*_*b*_ does not change with *ϕ*.

**Figure 12:**
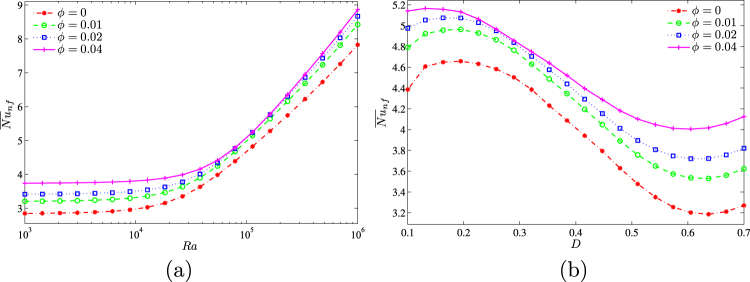
Variation of average Nusselt number with (**a**) *Ra* and (**b**) *D* for different *ϕ* at *Ha* = 15, *k*_*w*_ = 0.76 and *D* = 0.3.

Figure 12 shows dependences of the average Nusselt number on the Rayleigh number and nanoparticles volume fraction (Fig. 12a) and also centered solid block size and nanoparticles volume fraction (Fig. 12b). As has been mentioned above an increase in nanoparticles volume fraction leads to the heat transfer enhancement. The rate of this heat transfer enhancement depends on the solid block sizes.

**Figure 13:**
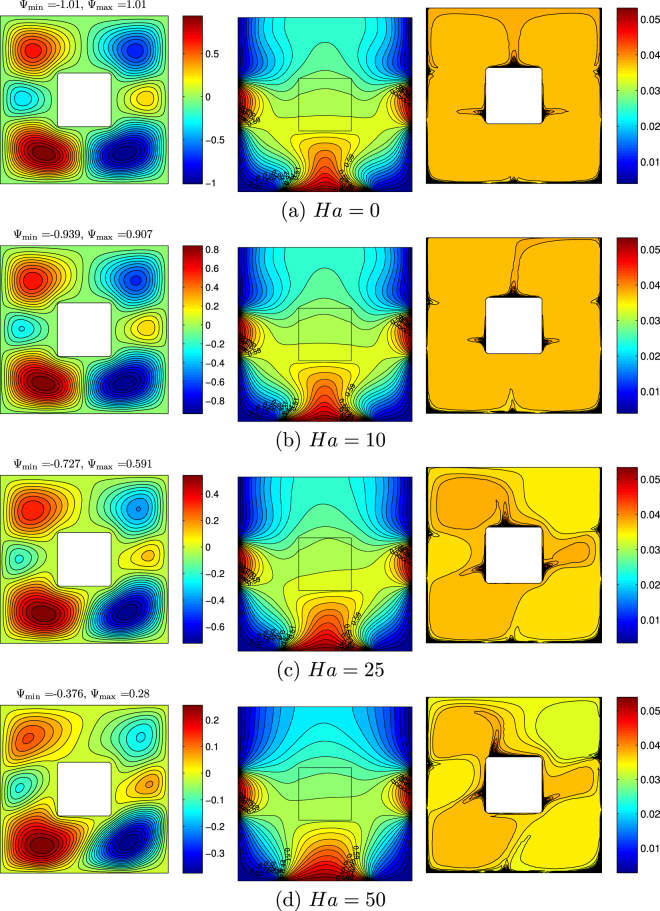
Variation of the streamlines (left), isotherms (middle), and nanoparticle distribution (right) evolution by Hartman number (*Ha*) for *Ra* = 10^5^, *ϕ* = 0.02, *k*_*w*_ = 0.76 and *D* = 0.3.

**Figure 14:**
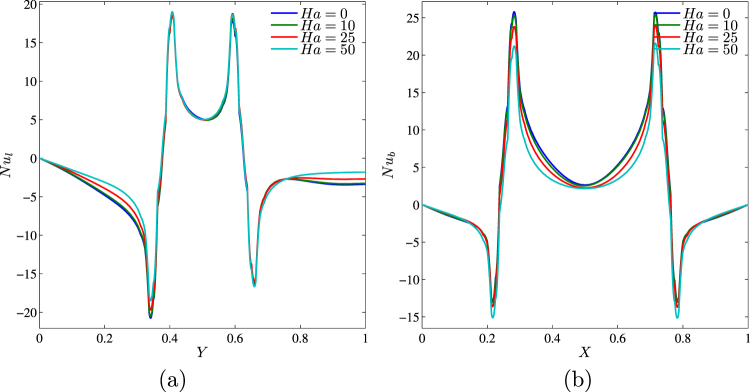
Variation of local Nusselt number interfaces with (**a**) *Y* and (**b**) *X* for different *Ha* at *Ra* = 10^5^, *ϕ* = 0.02, *k*_*w*_ = 0.76 and *D* = 0.3.

Figure 13 demonstrates streamlines, isotherms and isoconcentrations for different values of the Hartmann number at *Ra* = 10^5^, *ϕ* = 0.02, *k*_*w*_ = 0.76 and *D* = 0.3. It is well-known that in the case of MHD an increase in magnetic field intensity leads to the convective flow and heat transfer suppression. Therefore, in the present analysis a growth of *Ha* leads to the attenuation of convective flow and heat transfer rate reduction. Moreover, for high values of Hartmann number (>10) one can find a formation of asymmetric nanofluid flow structures, temperature and nanoparticles concentration fields due to an inclined influence of magnetic field where *γ* = *π*/4. Also for high values of Hartmann number isoconcentration field becomes non-homogeneous due to a domination of heat conduction and as a results an essential effect of thermophoresis. It is interesting to note a diagonal orientation of convective cells and thermal plume for high values of *Ha* (≥25). Namely, convective cells for *Ha* = 50 are elongated from left bottom corner till right upper corner and thermal plume for *Ha* = 25 also has the same orientation. Such behavior is related to the effect of inclined magnetic field where an inclination angle of the magnetic field is equal to *π*/4. The effect of the Hartmann number on the local Nusselt number (Fig. 14) illustrates a reduction of |*Nu*| with *Ha*.

**Figure 15:**
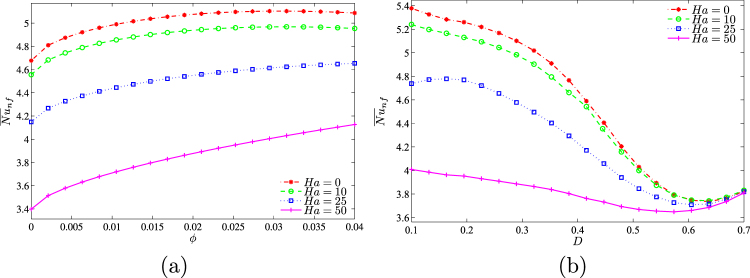
Variation of average Nusselt number with (**a**) *ϕ* and (**b**) *D* for different *Ha* at *Ra* = 10^5^, *k*_*w*_ = 0.76 and *D* = 0.3.

The average Nusselt number decreases with the Hartmann number (Fig. 15). Moreover, the heat transfer enhancement with the nanoparticles volume fraction is more essential for high values of the Hartmann number due to an intensification of the considered slip mechanisms for nanoparticles. At the same time, the maximum average Nusselt number at *ϕ* = 0.03 for *Ha* = 0 with a growth of the magnetic field intensity vanishes and the heat transfer rate becomes an increasing function of the nanoparticles volume fraction for high values of the Hartmann number. In the case of variations of the centered solid block size (Fig. 15b) one can find a significant decrease in ${\overline{Nu}}_{nf}$ with *Ha* for low values of block size, while an increase in *D* leads to a reduction of differences in the average Nusselt number between low and high values of the Hartmann number. In the case of *D* = 0.7 an increase in the Hartmann number does not change the heat transfer rate.

**Figure 16:**
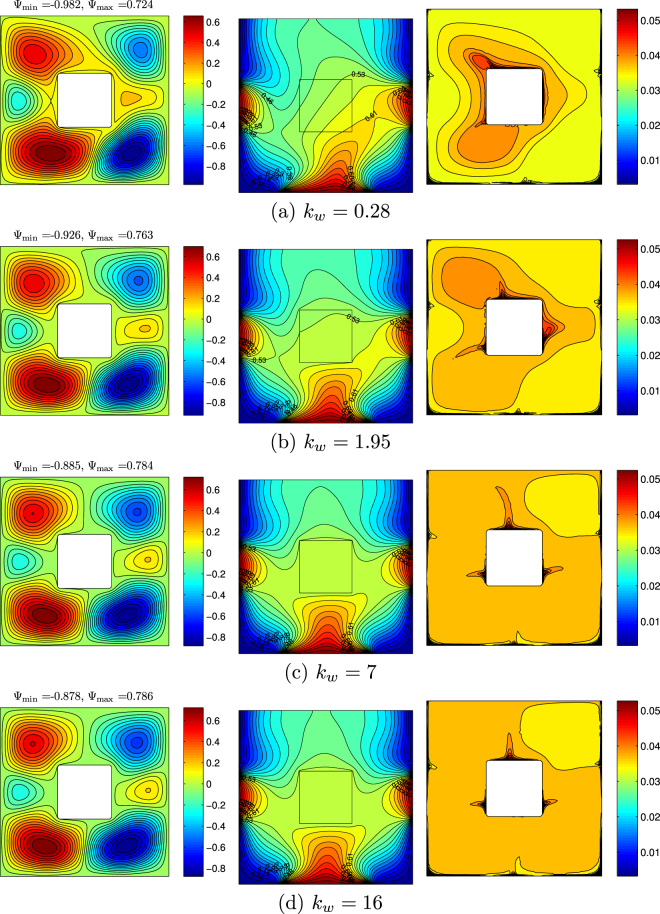
Variation of the streamlines (left), isotherms (middle), and nanoparticle distribution (right) evolution by thermal conductivity of the conductive inner block (*k*_*w*_) for *Ra* = 10^5^, *ϕ* = 0.02, *Ha* = 15 and *D* = 0.3.

Thermal conductivity ratio is a ratio between solid block thermal conductivity and nanofluid thermal conductivity. In the case of conjugate heat transfer problems this parameter plays an essential role, because it reflects a contribution of solid wall material in heat transfer process. Figure 16 illustrates streamlines, isotherms and isoconcentrations for different values of thermal conductivity ratio at *Ra* = 10^5^, *ϕ* = 0.02, *Ha* = 15 and *D* = 0.3. A growth of thermal conductivity ratio characterizes an increase in the thermal conductivity of solid block material, as a result this block is heated significantly from bottom and lateral heaters. In the case of *k*_*w*_ = 0.28 one can find an asymmetrical nanofluid flow structures and thermal plume over the bottom heater. Orientation of these thermo-hydrodynamic structures is under the effect of the inclined magnetic field. An increase in *k*_*w*_ leads to more essential heating of the solid block and a formation of symmetric convective cells and thermal plume over the bottom heater. At the same time, isoconcentrations illustrate more homogeneous distributions of nanoparticles for high values of thermal conductivity of solid block.

**Figure 17:**
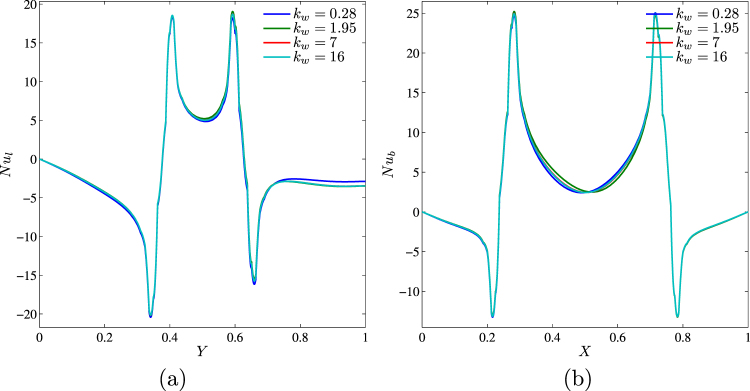
Variation of local Nusselt number interfaces with (**a**) *Y* and (**b**) *X* for different *k*_*w*_ at *Ra* = 10^5^, *ϕ* = 0.02, *Ha* = 15 and *D* = 0.3.

Profiles of the local Nusselt number along left vertical and bottom horizontal walls are shown in Fig. 17 for different values of the thermal conductivity ratio. Change of *k*_*w*_ leads to weak modification of *Nu*_*l*_ and *Nu*_*b*_.

**Figure 18:**
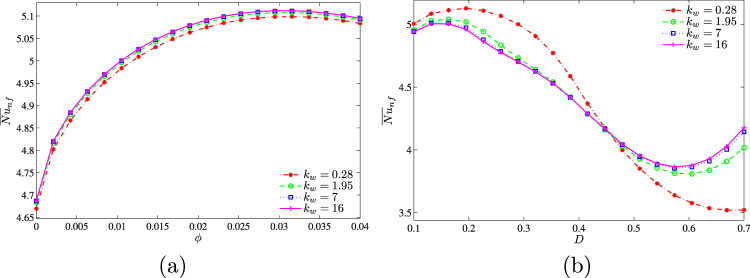
Variation of average Nusselt number with (**a**) *ϕ* and (**b**) *D* for different *k*_*w*_ at *Ra* = 10^5^, *Ha* = 15 and *D* = 0.3.

Figure 18 shows the variations of the average Nusselt number with *k*_*w*_,*ϕ* and *D*. An increase in kw (Fig. 18a) leads to a growth of ${\overline{Nu}}_{nf}$. In the case of different *D*, it is possible to highlight a non-linear effect of the thermal conductivity ratio for different values of the centered solid block sizes, namely, for *D* < 0.45 a growth of kw leads to the heat transfer rate reduction, while for *D* > 0.45 the effect is opposite.

**Figure 19:**
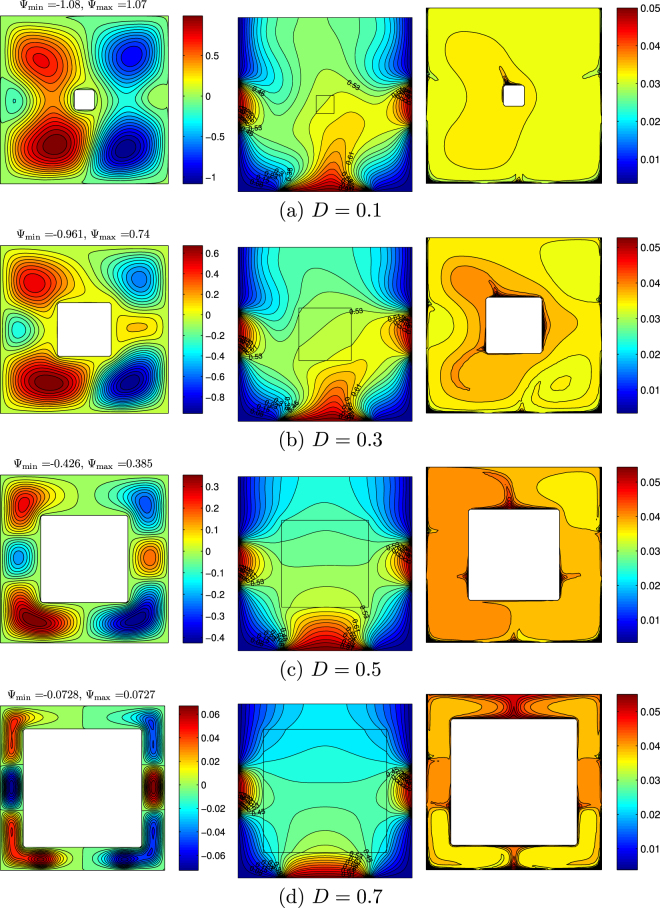
Variation of the streamlines (left), isotherms (middle), and nanoparticle distribution (right) evolution by length of the conductive inner block (*D*) for *Ra* = 10^5^, *ϕ* = 0.02, *Ha* = 15 and *k*_*w*_ = 0.76.

**Figure 20:**
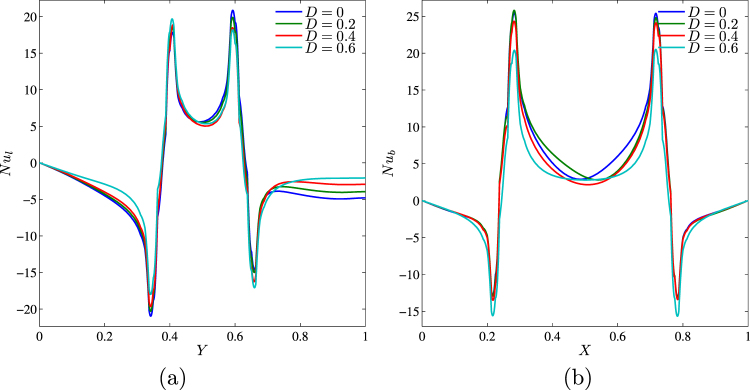
Variation of local Nusselt number interfaces with (**a**) *Y* and (**b**) *X* for different *D* at *Ra* = 10^5^, *ϕ* = 0.02, *Ha* = 15 and *k*_*w*_ = 0.76.

**Figure 21:**
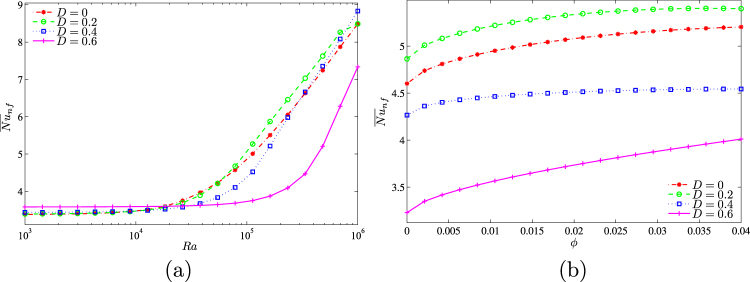
Variation of average Nusselt number with (**a**) *Ra* and (**b**) *ϕ* for different *D* at *Ha* = 15 and *k*_*w*_ = 0.76.

The effect of solid block size on streamlines, isotherms and nanoparticles isoconcentrations as well as local and average Nusselt numbers is demonstrated in Figs 19–21. Low volume (Fig. 19a,b) of the internal solid block characterizes a formation of asymmetric thermo-hydrodynamic structures with a homogeneous nanoparticles distributions. An increase in *D* leads to more essential deformation of solid structures that become symmetric with a thermal plume over the bottom heater. In the case of *D* = 0.7 (Fig. 19d) one can find several weak circulations inside a narrow gap between solid block and cavity walls. Behavior of the local Nusselt number does not change with *D* (Fig. 20). In the case of the average Nusselt number we can highlight a non-linear effect of the solid block size on the heat transfer enhancement (Fig. 21).

## Conclusions

In the present study, the finite difference method (FDM) is used to study the steady laminar MHD natural convection of an alumina-water nanofluid within a discretely heated square cavity with a centered heat-conducting solid block. The governing equations in dimensionless form have been formulated using the two-phase Buongiorno nanofluid model. The detailed computational results for the flow, temperature and nanoparticles volume fraction fields within the cavity, and the local and average Nusselt numbers are shown graphically for wide ranges of the Rayleigh number, Hartmann number, thermal conductivity ratio, solid block size and nanoparticles volume fraction. The important conclusions in the study are provided below:

1. High values of the Rayleigh number characterize more essential circulation inside the cavity. The distribution of nanoparticles becomes more homogeneous for high *Ra*.
2. The change of the nanoparticles volume fraction illustrates more essential modification of the nanoparticles isoconcentrations where more homogeneous distribution can be obtained for high values of *ϕ*.
3. The magnetic field intensity (the Hartmann number) suppresses the convective flow and heat transfer. The heat transfer enhancement with the nanoparticles volume fraction is more essential for high values of the Hartmann number. A significant decrease in ${\overline{Nu}}_{nf}$ with Ha can be found for low values of block size, while an increase in *D* leads to a reduction of differences in the average Nusselt number between low and high values of the Hartmann number. In the case of *D* = 0.7, an increase in the Hartmann number does not change the heat transfer rate.
4. An increase in the thermal conductivity ratio leads to more homogeneous distributions of nanoparticles. At the same time, for *D* < 0.45 a growth of kw leads to a heat transfer rate reduction, while for *D* > 0.45 the effect is the opposite.
5. The effect of the centered solid block size is non-linear on the heat transfer rate.

## References

[1] Ostrach S (1988). Natural convection in enclosures. J. Heat Transfer.

[2] Keyhani M, Dalton T (1996). Natural convection heat transfer in horizontal rod-bundle enclosures. Transactions-American Society Of Mechanical Engineers Journal Of Heat Transfer.

[3] Kaluri RS, Basak T (2011). Entropy generation due to natural convection in discretely heated porous square cavities. Energy.

[4] Kaluri RS, Basak T (2011). Role of entropy generation on thermal management during natural convection in porous square cavities with distributed heat sources. Chemical Engineering Science.

[5] Khanafer K, Vafai K, Lightstone M (2003). Buoyancy-driven heat transfer enhancement in a two-dimensional enclosure utilizing nanofluids. Int. J. Heat Mass Transfer.

[6] Sheikholeslami M, Ashorynejad H, Rana P (2016). Lattice Boltzmann simulation of nanofluid heat transfer enhancement and entropy generation. Journal of Molecular Liquids.

[7] Hu Y, He Y, Wang S, Wang Q, Schlaberg HI (2014). Experimental and numerical investigation on natural convection heat transfer of TiO_2_–water nanofluids in a square enclosure. J. Heat Transfer.

[8] Sheikholeslami M, Darzi M, Sadoughi M (2018). Heat transfer improvement and pressure drop during condensation of refrigerant-based nanofluid; an experimental procedure. International Journal of Heat and Mass Transfer.

[9] Sheremet MA, Pop I, Nazar R (2015). Natural convection in a square cavity filled with a porous medium saturated with a nanofluid using the thermal nonequilibrium model with a Tiwari and Das nanofluid model. International Journal of Mechanical Sciences.

[10] Alsabery AI, Saleh H, Hashim I, Siddheshwar PG (2016). Transient natural convection heat transfer in nanoliquid-saturated porous oblique cavity using thermal non-equilibrium model. International Journal of Mechanical Sciences.

[11] Alsabery, A., Chamkha, A., Saleh, H. & Hashim, I. Natural convection flow of a nanofluid in an inclined square enclosure partially filled with a porous medium. *Scientific Reports* **7** (2017). 10.1038/s41598-017-02241-x PMC5443814 28539585

[12] Sheikholeslami M, Seyednezhad M (2018). Simulation of nanofluid flow and natural convection in a porous media under the influence of electric field using CVFEM. International Journal of Heat and Mass Transfer.

[13] Corcione M (2011). Empirical correlating equations for predicting the effective thermal conductivity and dynamic viscosity of nanofluids. Energy Conversion and Management.

[14] Wen D, Ding Y (2004). Experimental investigation into convective heat transfer of nanofluids at the entrance region under laminar flow conditions. International Journal of Heat and Mass Transfer.

[15] Buongiorno J (2006). Convective transport in nanofluids. Journal of Heat Transfer.

[16] Hamid, R. A., Nazar, R. & Pop, I. Non-alignment stagnation-point flow of a nanofluid past a permeable stretching/shrinking sheet: Buongiorno’s model. *Scientific reports* **5** (2015). 10.1038/srep14640 PMC4594122 26440761

[17] Sheikholeslami M, Gorji-Bandpy M, Ganji D, Soleimani S (2014). Thermal management for free convection of nanofluid using two phase model. Journal of Molecular Liquids.

[18] Garoosi F, Rohani B, Rashidi MM (2015). Two-phase mixture modeling of mixed convection of nanofluids in a square cavity with internal and external heating. Powder Technology.

[19] Garoosi F, Hoseininejad F, Rashidi MM (2016). Numerical study of natural convection heat transfer in a heat exchanger filled with nanofluids. Energy.

[20] Motlagh SY, Soltanipour H (2017). Natural convection of Al_2_O_3_-water nanofluid in an inclined cavity using Buongiorno’s two-phase model. International Journal of Thermal Sciences.

[21] Nkurikiyimfura I, Wang Y, Pan Z (2013). Heat transfer enhancement by magnetic nanofluids—a review. Renewable and Sustainable Energy Reviews.

[22] Selimefendigil F, Öztop HF, Chamkha AJ (2016). MHD mixed convection and entropy generation of nanofluid filled lid driven cavity under the influence of inclined magnetic fields imposed to its upper and lower diagonal triangular domains. Journal of Magnetism and Magnetic Materials.

[23] Pirmohammadi M, Ghassemi M (2009). Effect of magnetic field on convection heat transfer inside a tilted square enclosure. International Communications in Heat and Mass Transfer.

[24] Mahmoudi AH, Pop I, Shahi M (2012). Effect of magnetic field on natural convection in a triangular enclosure filled with nanofluid. International Journal of Thermal Sciences.

[25] Ghasemi B, Aminossadati S, Raisi A (2011). Magnetic field effect on natural convection in a nanofluid-filled square enclosure. International Journal of Thermal Sciences.

[26] Kefayati GR (2013). Effect of a magnetic field on natural convection in an open cavity subjugated to water/alumina nanofluid using lattice Boltzmann method. International Communications in Heat and Mass Transfer.

[27] Sheikholeslami M, Gorji-Bandpy M, Ganji D (2013). Numerical investigation of mhd effects on Al_2_O_3_–water nanofluid flow and heat transfer in a semi-annulus enclosure using LBM. Energy.

[28] Sheikholeslami M, Bandpy MG, Ellahi R, Zeeshan A (2014). Simulation of mhd CuO–water nanofluid flow and convective heat transfer considering lorentz forces. Journal of Magnetism and Magnetic Materials.

[29] Selimefendigil F, Öztop HF (2015). Natural convection and entropy generation of nanofluid filled cavity having different shaped obstacles under the influence of magnetic field and internal heat generation. Journal of the Taiwan Institute of Chemical Engineers.

[30] Sheikholeslami M, Shehzad S (2018). Numerical analysis of Fe_3_O_4_-H_2_O nanofluid flow in permeable media under the effect of external magnetic source. International Journal of Heat and Mass Transfer.

[31] Sheikholeslami M, Shehzad S (2018). CVFEM simulation for nanofluid migration in a porous medium using darcy model. International Journal of Heat and Mass Transfer.

[32] Sivaraj C, Sheremet M (2017). MHD natural convection in an inclined square porous cavity with a heat conducting solid block. Journal of Magnetism and Magnetic Materials.

[33] Sheikholeslami M, Rokni HB (2018). Magnetic nanofluid flow and convective heat transfer in a porous cavity considering Brownian motion effects. Physics of Fluids.

[34] Sheikholeslami M (2018). CuO-water nanofluid flow due to magnetic field inside a porous media considering Brownian motion. Journal of Molecular Liquids.

[35] Kim DM, Viskanta R (1984). Study of the effects of wall conductance on natural convection in differently oriented square cavities. Journal of Fluid Mechanics.

[36] House JM, Beckermann C, Smith TF (1990). Effect of a centered conducting body on natural convection heat transfer in an enclosure. Numerical Heat Transfer.

[37] Ha MY, Jung MJ, Kim YS (1999). Numerical study on transient heat transfer and fluid flow of natural convection in an enclosure with a heat-generating conducting body. Numerical Heat Transfer: Part A: Applications.

[38] Zhao F-Y, Liu D, Tang G-F (2007). Conjugate heat transfer in square enclosures. Heat and Mass Transfer.

[39] Mahmoodi M, Sebdani SM (2012). Natural convection in a square cavity containing a nanofluid and an adiabatic square block at the center. Superlattices and Microstructures.

[40] Mahapatra PS, De S, Ghosh K, Manna NK, Mukhopadhyay A (2013). Heat transfer enhancement and entropy generation in a square enclosure in the presence of adiabatic and isothermal blocks. Numerical Heat Transfer, Part A: Applications.

[41] Alsabery AI, Siddheshwar PG, Saleh H, Hashim I (2016). Transient free convective heat transfer in nanoliquid-saturated porous square cavity with a concentric solid insert and sinusoidal boundary condition. Superlattices and Microstructures.

[42] Garoosi F, Rashidi MM (2017). Two phase flow simulation of conjugate natural convection of the nanofluid in a partitioned heat exchanger containing several conducting obstacles. International Journal of Mechanical Sciences.

[43] Maxwell, J. C. *A Treatise on Electricity and Magnetism, vol. II. Clarendon* (Oxford University Press 1904).

[44] Ho C, Liu W, Chang Y, Lin C (2010). Natural convection heat transfer of alumina-water nanofluid in vertical square enclosures: An experimental study. International Journal of Thermal Sciences.

[45] Sheikhzadeh GA, Dastmalchi M, Khorasanizadeh H (2013). Effects of nanoparticles transport mechanisms on Al_2_O_3_-water nanofluid natural convection in a square enclosure. International Journal of Thermal Sciences.

[46] Corcione M, Cianfrini M, Quintino A (2013). Two-phase mixture modeling of natural convection of nanofluids with temperature-dependent properties. International Journal of Thermal Sciences.

[47] Sheremet MA, Pop I (2014). Conjugate natural convection in a square porous cavity filled by a nanofluid using Buongiorno’s mathematical model. International Journal of Heat and Mass Transfer.

[48] Bergman, T. L. & Incropera, F. P. *Introduction to heat transfer, 6 th edition* (New York: Wiley 2011).

[49] Chon CH, Kihm KD, Lee SP, Choi SU (2005). Empirical correlation finding the role of temperature and particle size for nanofluid (Al_2_O_3_) thermal conductivity enhancement. Applied Physics Letters.

